# Amplified P-wave duration predicts incident atrial fibrillation in the general population: Results from the Hamburg City Health Study

**DOI:** 10.1016/j.hroo.2026.03.033

**Published:** 2026-04-02

**Authors:** Lena R. Koch, Silvia Becker, Ana Bošnjak, Marie Biedermann, Amir S. Jadidi, Dirk Westermann, Heiko Lehrmann, Axel Loewe, Thomas Arentz, Maximilian Schell, Götz Thomalla, Daniel Engler, Renate B. Schnabel, Martin Eichenlaub

**Affiliations:** 1 Department of Cardiology and Angiology, University Medical Center, Faculty of Medicine, University of Freiburg, Bad Krozingen, Germany; 2 Institute of Biomedical Engineering, Karlsruhe Institute of Technology, Karlsruhe, Germany; 3 Department of Cardiology, Heart Center Lucerne – Lucerne Cantonal Hospital, Lucerne, Switzerland; 4 Department of Neurology and; 5 Department of Cardiology, University Heart and Vascular Center Hamburg, University Medical Center Hamburg-Eppendorf, Hamburg, Germany; 6 German Center for Cardiovascular Research, Partner Site Hamburg/Kiel/Lübeck, Hamburg, Germany

**Keywords:** Atrial fibrillation, Screening, Electrocardiography, Risk assessment, Preventive cardiology

## Abstract

**Background:**

Clinical risk factors identify subjects at risk of incident atrial fibrillation (AF) with only mediocre accuracy. Amplified P-wave duration (PWD) is a novel electrocardiographic (ECG) marker for conduction delay in atrial cardiomyopathy, which in turn is associated with AF.

**Objective:**

This study aimed to assess whether amplified PWD can predict incident AF in the general population.

**Methods:**

Amplified PWD was assessed manually on a signal-modified ECG in 2134 participants from the population-based Hamburg City Health Study. Incident AF during the 5-year follow-up was defined as the primary end point. Multivariable Cox regression models were constructed including significant clinical risk factors, ECG parameters, and transthoracic echocardiography parameters, and the predictive power for the primary end point was evaluated.

**Results:**

Over a median follow-up of 5.5 years, 102 participants (4.8%) developed incident AF. The upper tertile of amplified PWD was the only significant ECG-derived predictor for incident AF (hazard ratio [HR] 2.02; *P* = .010) in the multivariable ECG-based model, alongside age (HR 1.07; *P* = .013) and body mass index (HR 1.08; *P* = .003), after adjustment for significant clinical risk factors (concordance index 0.764). A comprehensive combined ECG/transthoracic echocardiography model achieved a concordance index of 0.770, with age (HR 1.07; *P* = .020) and left atrial volume (HR 1.02; *P* = .020) remaining significant predictors.

**Conclusion:**

Amplified PWD as a novel ECG-based marker predicts incident AF in a population-based cohort and may provide a pragmatic ECG-derived marker of atrial cardiomyopathy for risk assessment beyond conventional risk stratification.

**Clinical Trial Registration:**

NCT03934957 (https://www.clinicaltrials.gov)

**Key Findings**

▪ Amplified P-wave duration as novel electrocardiogram-based biomarker predicts incident atrial fibrillation in the general population.
▪ Prolonged amplified P-wave duration is significantly associated with new-onset atrial fibrillation during a 5-year follow-up period.
▪ Incorporating amplified P-wave duration improves risk stratification by the CHA_2_DS_2_-VA score for incident atrial fibrillation.

## Introduction

Atrial fibrillation (AF) and ischemic stroke are 2 closely related conditions with increasing prevalence and substantial socioeconomic burden.^1^^,^^2^ Fortunately, both conditions are amenable to a wide range of preventive and therapeutic options. Thus, early identification of individuals at risk of incident AF presents an opportunity to mitigate disease burden and improve outcomes.

Most commonly, disease prediction models rely on readily available clinical parameters for risk stratification. The CHA_2_DS_2_-VA score is the most widely used tool to predict the risk of ischemic stroke in patients with established AF, and its use has recently been extended for the prediction of incident AF and ischemic stroke in subjects without AF.^3^^,^^4^ By aggregation of clinical parameters, the CHA_2_DS_2_-VA score achieves moderate accuracy at minimal cost, yet, it lacks the granularity required for risk prediction on an individual level.^5^

Complementary to clinical risk prediction models, electrocardiography (ECG)-based markers, such as P-wave terminal force in V1 (PTFV1), P-wave duration (PWD), and advanced interatrial block (IAB), are emerging as valuable tools for the prediction of incident AF and ischemic stroke.^6^, ^7^, ^8^ This is grounded on the rationale that the P wave captures electrical and mechanical remodeling processes of the atria, which form the substrate for the manifestation of AF and the formation of atrial thrombi causing cardioembolic stroke.^9^

PWD reflects electrical conduction time across the atria and is prolonged in the setting of atrial remodeling. Of note, several studies report a nonlinear, parabolic connection with both abnormally long and short PWDs associated with AF and stroke.^10^^,^^11^ Our group has recently developed a novel method for measuring PWD based on a signal-amplified P-wave visualization. This technique specifically preserves low-amplitude parts of the P wave as a surrogate for advanced atrial remodeling, enabling a precise measurement without signal degradation.^12^^,^^13^ Analysis of amplified PWD demonstrated superior performance to conventional measurements of PWD in identifying patients with AF based on their sinus rhythm ECG.^14^ However, the application of amplified PWD as a noninvasive screening tool in the general population to identify individuals at risk is still pending.

This study aimed to evaluate the predictive utility of amplified PWD for incident AF in the Hamburg City Health Study, a large population-based cohort study.

## Methods

### Study population

The Hamburg City Health Study is a cohort project initiated by physicians and scientists from the University Medical Center Hamburg-Eppendorf. In total, approximately 45,000 participants were recruited from the Hamburg general population aged 45–74 years by random sampling from the official inhabitant data file.^15^ Recruitment took place between 2016 and 2022. An extensive description of the Hamburg City Health Study’s design and methodology has been published previously.^15^

**Figure 1:**
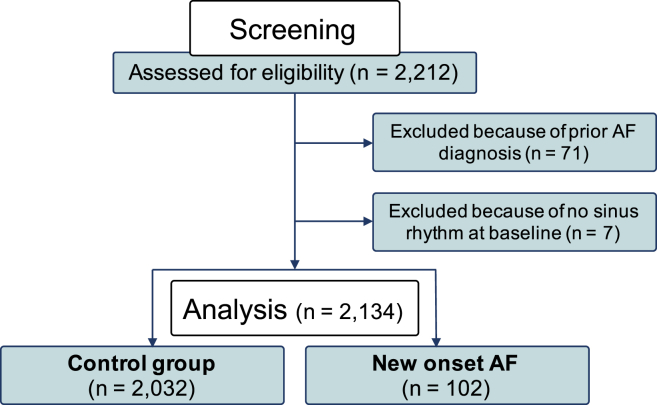
Participant flowchart. Subjects from the Hamburg City Health Study were included in the analysis based on the outlined criteria. AF = atrial fibrillation.

Participants underwent a comprehensive baseline assessment including anthropometric measures, echocardiography, and digital 12-lead ECG. During the first follow-up, medical records on major medical events including the time point of diagnosis were gathered. At the time of the present analysis, complete longitudinal follow-up information required for the current investigation was available for approximately 10,000 participants. From this population, we analyzed the first 2212 consecutively included participants with complete baseline digital ECG recordings and follow-up information without additional clinical preselection. After exclusion of participants with previous AF diagnosis and no sinus rhythm at baseline (Figure 1), the final study sample comprised 2134 participants.

The study complied with the ethical principles of the Declaration of Helsinki and was approved by the local ethics committee of the Landesaerztekammer Hamburg (State of Hamburg Chamber of Medical Practitioners, PV5131) and by the Data Protection Commissioner of the University Medical Center of the University Hamburg-Eppendorf and the Data Protection Commissioner of the Free and Hanseatic City of Hamburg. The study has been registered at ClinicalTrial.gov (NCT03934957), and all participants provided a written informed consent before enrolment.

### Amplified P-wave analysis

A digital 12-lead ECG was obtained for all participants at baseline using CARDIOVIT CS-200 Excellence, and conventional, nonamplified PWD and PTFV1 were computed automatically by SEMA Schiller AT-104 PC software (both from Schiller Medizintechnik GmbH, Baar, Switzerland).

**Figure 2:**
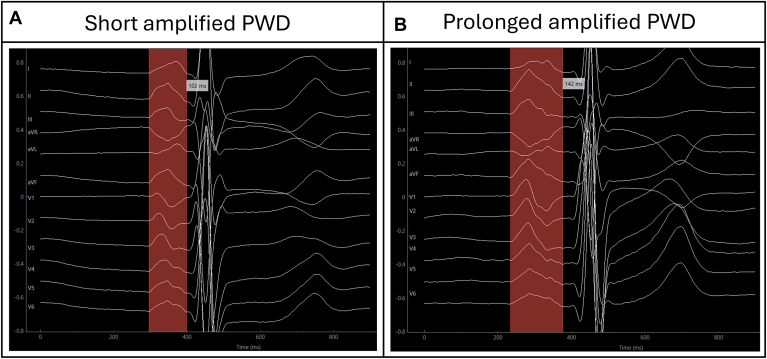
Illustration of amplified PWD measurement. Digital 12-lead electrocardiogram recordings were automatically signal-averaged and displayed using amplified scaling (gain 80 mm/mV; sweep speed 175 mm/s). **A:** An example of a short amplified PWD (102 ms). **B:** A prolonged amplified PWD (142 ms). *Vertical lines* of the *red-shaded area* indicate the earliest onset and the latest offset of the amplified PWD across all leads used for measurement. PWD = P-wave duration.

For amplified PWD analysis, ECG raw data were used without further noise filtering to generate an averaged beat for each ECG lead to eliminate beat-to-beat P-wave variances and enhance the signal-to-noise ratio. Signal amplification was performed at a gain of 80 mm/mV and a sweep speed of 175 mm/s for optimal visualization of late, low-amplitude components of the P wave. Further information on the ECG amplification method has been published previously.^12^ Amplified PWD was measured from the earliest initial deflection in any lead to the latest end in any lead and typically requires only a few seconds per ECG once the operator is trained. An example of the software interface illustrating amplified PWD measurement, including representative examples of a short and a prolonged P wave, is presented in Figure 2.

Inferior leads II, III, and aVF were further evaluated for biphasicity (positive-negative deflection), and advanced IAB was defined as the presence of a biphasic P wave in all inferior leads. All measurements were performed by 2 trained physicians blinded to all clinical and demographic information and outcome-related data.

### Outcome

The primary end point consists of incident AF during follow-up. Diagnosis of incident AF was based on self-statements and medical records. In addition, we defined a composite end point consisting of incident AF and ischemic stroke during follow-up. Ischemic stroke events were identified based on self-reported information and medical records. In cases of 2 consecutive events, only the first event was considered in the time-to-event analysis. Because ischemic stroke events were not systematically adjudicated by an independent end point committee and information on stroke etiology (eg, cardioembolic vs noncardioembolic) was incomplete, all stroke-related analyses are presented exclusively in the Supplemental Material.

### Statistical methods

Continuous data are displayed as median with first and third quartiles (Q1, Q3) or mean ± standard deviation and compared using the Student *t* test. Categorical data are summarized as absolute counts and percentages, with group comparisons performed using the χ^2^ test.

Time-to-event survival probabilities were computed using the Kaplan-Meier method. In case the exact date of an end point event was unknown, its time point was approximated using mean imputation.

Univariable and multivariable Cox regression analyses were used to assess the association between clinical risk factors, ECG- and transthoracic echocardiography (TTE)–based parameters, and the primary end point. We constructed different multivariable model specifications to address potential confounding and to evaluate the incremental value of amplified PWD in relation to structural atrial remodeling:

(1) An ECG-based multivariable model including clinical risk factors and ECG parameters
(2) A TTE-based multivariable model including clinical risk factors and LA volume
(3) A combined ECG/TTE model including clinical risk factors, ECG parameters, and LA volume

Results are reported as HRs and 95% confidence intervals (CIs). A potential nonlinear relationship between PWD and the primary end point was assessed more in-depth with a Cox regression using polynomial splines. When analysis did not reveal a significant nonlinear component for amplified PWD, only linear effects of amplified PWD were assessed in further Cox regression. Model performance was evaluated using the Akaike information criterion and the concordance index (C-index). Nested Cox models were compared by the likelihood ratio test.

Exploratory stroke analyses (Supplemental Material) were assessed separately owing to limited event numbers and lack of systematic adjudication.

All tests with *P* < .05 were considered significant. Data processing and statistics were performed in R version 4.3.

## Results

### Demographic information at baseline

A total of 2134 subjects from the Hamburg City Health Study cohort were analyzed. During a median follow-up of 5.5 years (Q1–Q3 4.5–6.0 years), incident AF was diagnosed in 102 participants (4.8%). The median time to AF diagnosis was 3.7 years (Q1–Q3 2.1–4.8 years).

**Figure 3:**
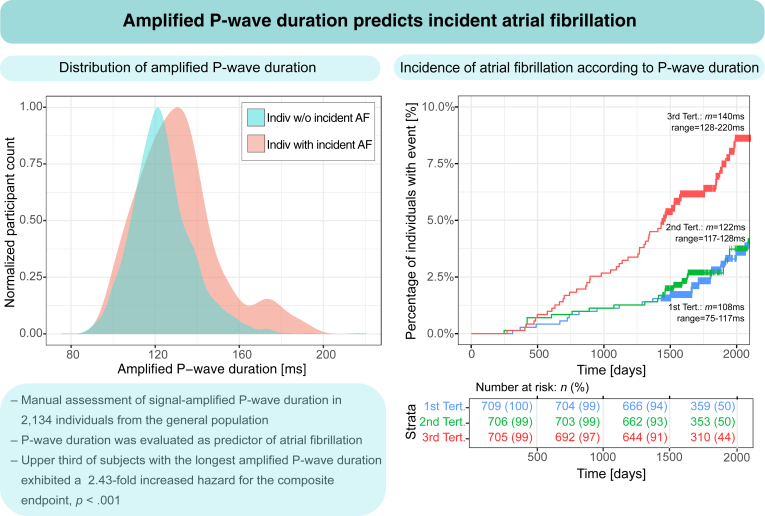
Frequency distribution of amplified PWD and incidence of atrial fibrillation stratified by PWD. Left: The frequency distribution of amplified PWD for participants with (*red*) and without (*turquoise*) an end point event. Participant count was normalized to the respective maximum frequency. Right: Kaplan-Meier event curves for the primary end point with participants split into 3 bins based on their amplified PWD. Results are displayed in *blue*, *green*, and *red* for participants with the shortest, intermediate, and longest amplified PWD. Per bin, amplified PWD range and mean are displayed; the table displays the count of participants at risk of the end point, with percentages given in *brackets*. AF = atrial fibrillation; Indiv = individuals; m = mean; PWD = P-wave duration; Tert = tertile; w/o = without.

Participants who developed incident AF were significantly older (69.5 vs 65.4 years; *P* < .001) and had a higher body mass index (28.5 vs 26.5 kg/m^2^; *P* < .001). Coronary artery disease (14.0% vs 6.1%; *P* = .001), heart failure (12.7% vs 2.5%; *P* < .001), and a higher CHA_2_DS_2_-VA score (1.58 vs 1.00; *P* < .001) were more frequent among participants with incident AF (Table 1). Left atrial volume was significantly larger in participants who developed AF (62.4 vs 50.3 mL; *P* < .001). Regarding ECG parameters, amplified PWD was significantly longer in individuals who developed AF (130.9 vs 123.5 ms; *P* < .001) (Figure 3, left panel). The automatically computed PWD calculated by the Schiller algorithm showed only a modest difference (119.4 vs 116.1 ms; *P* = .017). Advanced IAB was more prevalent among AF cases (6.9% vs 1.4%; *P* = .001), whereas PTFV1 did not differ significantly between groups.

**Table 1:** Participant demographic and clinical characteristics

Characteristics	All (N = 2134)	No AF (n = 2032)	Incident AF (n = 102)	*P* value
Age (y)	65.60 ± 7.71	65.40 ± 7.76	69.47 ± 5.47	<.001∗
Male sex	983 (46)	927 (46)	56 (55)	.066
Body mass index (kg/m^2^)	26.64 ± 4.39	26.54 ± 4.32	28.49 ± 5.39	<.001∗
Diabetes mellitus	148 (7.1)	139 (7.0)	9 (9.0)	.50
Smoking history	1358 (64)	1290 (64)	68 (67)	.50
Coronary artery disease	137 (6.5)	123 (6.1)	14 (14)	.001∗
Myocardial infarction	86 (4.0)	78 (3.9)	8 (7.8)	.064
Heart failure				<.001∗
HFpEF	30 (2.1)	20 (1.5)	10 (13)	
HFmrEF	30 (2.1)	27 (2.0)	3 (4.0)	
HFrEF	4 (0.3)	4 (0.3)	0 (0)	
CHA_2_DS_2_-VA score	1.03 ± 0.91	1.00 ± 0.89	1.58 ± 1.08	<.001∗
LVEF (%)	58.63 ± 4.95	58.66 ± 4.95	58.04 ± 5.00	.30
Left atrial volume (mL)	50.93 ± 16.81	50.25 ± 16.18	62.35 ± 22.36	<.001∗
ECG markers				
Amplified P-wave duration (ms)	123.80 ± 15.23	123.45 ± 14.90	130.87 ± 19.47	<.001∗
Automated P-wave duration (ms)	116.29 ± 16.07	116.13 ± 15.38	119.38 ± 26.24	.017∗
Advanced interatrial block	36 (1.7)	29 (1.4)	7 (6.9)	.001∗
P-wave terminal force in V1 (mV × ms)	2395 ± 2065	2396 ± 2058	2382 ± 2213	.80

Values are presented as mean ± standard deviation and number (percentage).

AF = atrial fibrillation; CHA_2_DS_2_-VA = congestive heart failure, hypertension, age ≥75, diabetes mellitus, stroke, vascular disease, and age ≥65; ECG = electrocardiogram; HFmrEF = heart failure with mildly reduced ejection fraction; HFpEF = heart failure with preserved ejection fraction; HFrEF = heart failure with reduced ejection fraction; LVEF = left ventricular ejection fraction.

∗ Statistically significant.

### Follow-up

Kaplan-Meier estimates analyzing amplified PWD as a predictor for the primary end point are presented in the right panel of Figure 3. HRs are presented separately for each participant tertile with the shortest, intermediate, and longest amplified PWD. Univariable Cox regression demonstrated that longer amplified PWDs were associated with the primary end point with a 2% risk increase per 1 ms increment in PWD (95% CI 1.02–1.03; *P* < .001). The tertile of subjects with the longest amplified PWD (≥128 ms) exhibited a 143% risk increase (95% CI 1.65–3.59; *P* < .001) compared with the remainder of the cohort.

The upper tertile of amplified PWD remained the only independent ECG-derived predictor of incident AF in a multivariate ECG-based Cox regression model incorporating ECG parameters in addition to significant clinical risk factors with a 2.02-fold increased hazard (95% CI 1.18–3.45; *P* = .010) (Table 2). Age and body mass index also remained significant predictors, and the model achieved a C-index of 0.764.

**Table 2:** Multivariable Cox proportional hazards models with amplified PWD predicting atrial fibrillation

Variable	ECG model		Echo model		Combined model	
	HR (95% CI)	*P* value	HR (95% CI)	*P* value	HR (95% CI)	*P* value
Age	1.07 (1.01–1.13)	.013∗	1.08 (1.02–1.14)	.006	1.07 (1.01–1.13)	.020∗
BMI (kg/m^2^)	1.08 (1.03–1.14)	.003∗	1.05 (0.99–1.11)	.13	1.05 (0.99–1.12)	.11
CHA_2_DS_2_-VA score	1.37 (1.00–1.88)	.050	1.24 (0.88–1.76)	.20	1.30 (0.91–1.87)	.2
Heart failure	1.52 (0.70–3.28)	.3	1.92 (0.89–4.15)	.10	1.54 (0.68–3.49)	.3
Left atrial volume (mL)			1.02 (1.01–1.04)	.001∗	1.02 (1.00–1.03)	.020∗
Amplified PWD, upper tertile	2.02 (1.18–3.45)	.010∗			1.60 (0.88–2.89)	.12
Automated PWD, upper tertile	1.05 (0.61–1.80)	.9			1.06 (0.59–1.90)	.8
Advanced interatrial block	2.60 (0.80–8.48)	.11			3.04 (0.91–10.2)	.072
P-wave terminal force in V1 (mV × ms)	1.00 (1.00–1.00)	.3			1.00 (1.00–1.00)	.11
Model parameters						
Likelihood ratio test *P* value	<.001∗		<.001∗		<.001∗	
AIC	879		787		723	
C-index	0.764		0.751		0.770	

AIC = Akaike information criterion; BMI = body mass index; CHA_2_DS_2_-VA = congestive heart failure, hypertension, age ≥75, diabetes mellitus, stroke, vascular disease, and age ≥65; CI = confidence interval; C-index = concordance index; ECG = electrocardiogram; HR = hazard ratio; PWD = P-wave duration.

∗ Statistically significant.

To evaluate the independent association of amplified PWD with incident AF in relation to structural remodeling, we compared different multivariable model specifications. A comprehensive combined ECG/TTE model including clinical risk factors, ECG parameters, and left atrial volume achieved a C-index of 0.770. In this fully adjusted model, age and left atrial volume remained independently associated with incident AF (Table 2).

A TTE-based model including clinical risk factors and left atrial volume alone yielded a C-index of 0.751, with age and left atrial volume remaining significant predictors (Table 2).

### Comparison of amplified and automated PWD

**Figure 4:**
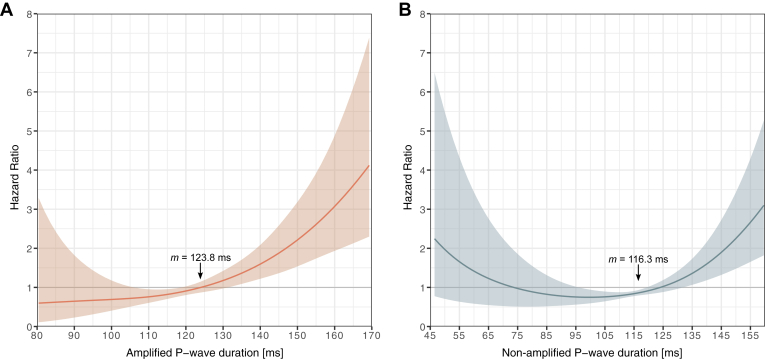
Hazard ratio for incident atrial fibrillation according to PWD. **A:** The hazard ratio of the amplified PWD relative to a baseline hazard for the mean of 124 ms in the study population. **B:** The hazard ratio of the automated PWD relative to a baseline hazard for the mean of 116 ms in the study population. m = mean; PWD = P-wave duration.

To evaluate a possible nonlinear relationship between amplified PWD and the primary end point, Cox regression with polynomial splines was performed. The analysis revealed a linear relationship with a risk increase with longer amplified PWDs (linear effect *P* < .001; nonlinear effect *P* = .32) (Figure 4A). Of note, previous literature reports a parabolic relationship between automatically assessed PWD and AF, where extremely short P waves are also associated with an increased risk of AF.^10^ Therefore, we reperformed the previous analysis with the automated PWD computed by the Schiller algorithm, which revealed an increased risk of both abnormally short and prolonged automated PWDs (linear effect *P* = .36; nonlinear effect *P* < .001) (Figure 4B). We hypothesized that these divergent observations for short PWDs were caused by a lower performance of the Schiller algorithm to recognize late, low-amplitude components of the P wave and biphasic P waves. To follow up this hypothesis, we focused on the 5% of participants with the shortest automated PWDs (Supplemental Table A.1). Although in the overall study population, amplified and automated PWD differed by a mean of 7.6 ± 16.9 ms, in the 5% of individuals with the shortest automated PWDs, the corresponding amplified PWDs were 35.3 ms longer (*P* < .001) (Supplemental Figure A.1A and A.1B). Inversely, the corresponding automated PWDs in the 5% of individuals with shortest amplified PWDs were on average 1.3 ms shorter (*P* = .424) (Supplemental Figure A.1C).

### Amplified PWD and risk of incident AF and ischemic stroke

In exploratory analyses, we evaluated the association between amplified PWD and the composite end point of incident AF and ischemic stroke. After exclusion of individuals with previous stroke or anticoagulant therapy at baseline, the composite end point occurred in 126 of 2054 participants (6.1%) during follow-up. Given that the analysis was restricted to the first occurrence of either event, the end point comprised 90 events of AF and 36 events of ischemic stroke.

Participants in the highest tertile of amplified PWDexhibited a significantly increased risk compared with the remainder of the cohort (HR 1.95; 95% CI 1.38–2.77; *P* < .001).Analyzing the contribution of each end point component revealed that the effect was mainly driven by the association between amplified PWD and incident AF (HR 1.03; 95% CI 1.01–1.04; *P* < .001), whereas there was no significant association between amplified PWD and ischemic stroke (HR 1.00; 95% CI 0.99–1.02; *P* = .75) (Supplemental Figure A.2).

## Discussion

Early identification of people at risk of incident AF is of considerable relevance as (1) the risk of AF is modifiable through interventions such as lifestyle modifications, (2) subjects at risk can undergo a targeted screening, and (3) a timely treatment initiation can avert disease complications and promote treatment success. In the present study, we demonstrated that amplified PWD as a noninvasive ECG-based diagnostic tool predicts incident AF during follow-up in a population-based cohort from the Hamburg City Health Study. Amplified PWD provides incremental predictive value beyond clinical risk stratification and is superior to other established P-wave indices in the prediction of incident AF.

### PWD as a predictor for incident AF

Here, we find that the upper tertile of amplified PWD is associated with a 143% risk increase for incident AF. Previous literature demonstrated an association between a prolonged P wave and incident AF for various patient cohorts with previous cardiovascular disease.^7^^,^^16^^,^^17^ Although these studies were performed in high-risk populations, our results indicate that amplified PWD also serves as a useful screening marker for AF in the general population.

Surprisingly, some studies on PWD report also an increased risk of AF in subjects with markedly short PWDs.^10^^,^^11^ Although prolonged PWDs reflect atrial conduction delay owing to atrial dilation and fibrotic tissue transformation, it remains poorly understood why abnormally short PWDs would be associated with an increased risk of AF. In the present analysis, we were able to replicate this association when PWD was assessed using automated measurements. Notably, the association vanished when PWD was determined using a signal-amplified P-wave visualization designed to preserve low-amplitude parts of the P wave. This may suggest that P-wave changes associated with relevant atrial remodeling are inadequately captured by conventional PWD measurement techniques. Although absolute differences in amplified PWD between participants with and without incident AF were modest, such small differences are expected in a predominantly healthy, population-based cohort with early or subclinical atrial disease. The clinical relevance of amplified PWD lies less in large absolute separation and more in its ability to unmask conduction abnormalities that may remain undetected with conventional automated measurements, thereby improving risk stratification in ranges where standard PWD has limited discriminatory power.

Reports on the association between PWD and ischemic stroke are more heterogeneous.^18^^,^^19^ Only recently, a systematic review reported no association between prolonged PWD and ischemic stroke for most studies on the matter.^20^ In line with this, amplified PWD does not predict the occurrence of ischemic stroke in this subpopulation from the Hamburg City Health Study. However, given the limited number of ischemic stroke events and the lack of systematic adjudication of stroke etiology, these findings should be interpreted cautiously.

### ECG and TTE markers of atrial remodeling

In comparison with amplified PWD, advanced IAB and PTFV1 are more established P-wave indices associated with an increased risk of AF, AF recurrences, and stroke.^6^, ^7^, ^8^^,^^16^^,^^21^ However, advanced IAB is a relatively rare ECG finding, which typically manifests only in advanced stages of atrial remodeling, limiting its utility as an early predictive marker. PWD presents a continuous ECG parameter, for which a prolongation can already be observed during early stages of atrial remodeling.^8^^,^^12^^,^^13^ In our analysis, amplified PWD was the only ECG-derived marker that remained independently associated with incident AF after adjustment for relevant clinical risk factors in an ECG-based multivariable model, whereas automated PWD, advanced IAB, and PTFV1 did not retain independent significance.

Of note, PWD differs from clinical risk factors given that it is less of a risk factor than an early marker for incipient atrial remodeling. In line with this, PWD correlates with atrial low-voltage areas, reduced atrial contractility, and episodes of AF.^22^ Thus, amplified PWD may complement clinical risk stratification by identifying individuals in whom atrial remodeling processes are already present.

Left atrial volume, assessed by TTE, was likewise a strong predictor of incident AF in the present study and remained independently associated in the comprehensive combined ECG/TTE model. The attenuation of the association between amplified PWD and incident AF after adjustment for left atrial volume suggests partial overlap between electrical and structural markers of atrial remodeling.

Our findings indicate that both left atrial volume and amplified PWD represent relevant surrogate markers of atrial cardiomyopathy, reflecting complementary aspects of structural and electrical remodeling. Although TTE provides a direct assessment of atrial size and structure, an ECG-derived marker such as amplified PWD may offer practical advantages owing to the wide availability, standardization, cost-effectiveness, and operator independence of ECG compared with TTE. Accordingly, amplified PWD may provide clinically useful information, particularly in settings where TTE is not routinely available, and may represent a pragmatic and scalable marker for routine ECG-based risk stratification.

### Limitations

This study used data from a prospective cohort study, and limitations arise from the observational nature of the trial. Incident AF was ascertained based on self-report and available medical records. Therefore, undetected asymptomatic AF cannot be excluded. Ischemic stroke events were not systematically adjudicated by an independent end point committee, and information on stroke etiology was incomplete. Accordingly, stroke analyses are underpowered and therefore presented exclusively as exploratory analyses in the Supplemental Material.

A limitation of amplified PWD is that it depends on manual measurement of the P wave, which may introduce observer variability and may hinder a widespread integration into clinical practice. Each P wave was measured by only 1 physician so that no data on inter-rater reliability are available. However, investigators were blinded to any clinical data, and previous studies had demonstrated a low interobserver variability.^12^ Nevertheless, automated measurement algorithms will be required for scalability and broader clinical implementation.

## Conclusion

Amplified PWD as a novel ECG-based marker predicts incident AF in a population-based cohort and might offer additional prognostic value beyond conventional risk stratification using clinical risk prediction models. Therefore, amplified PWD may serve as a widely applicable screening tool to facilitate early identification of individuals at risk of AF and potentially prevent adverse clinical outcomes.

## Disclosures

R.B.S. has received funding from the European Research Council under the European Union’s Horizon 2020 research and innovation program under the grant agreement no. 648131, the European Union’s Horizon 2020 research and innovation program under the grant agreement no. 847770 (AFFECT-EU), the European Union’s Horizon Europe research and innovation program under the grant agreement ID 101095480, German Center for Cardiovascular Research (DZHK e.V.) (81Z1710103 and 81Z0710114), German Ministry of Research and Education (BMBF 01ZX1408A), ERACoSysMed3 (031L0239), and Wolfgang Seefried project funding from the German Heart Foundation. R.B.S. has received lecture fees and advisory board fees from Bristol Myers Squibb/Pfizer and Bayer outside this work. D.W. received honoraria from Abiomed, AstraZeneca, Edwards, Meril, and Novartis outside the submitted work. The remaining authors have nothing to disclose.

## Appendix Supplementary Data

Supplementary Table

Figure A1

Figure A2

## References

[1] Lippi G., Sanchis-Gomar F., Cervellin G. (2021). Global epidemiology of atrial fibrillation: an increasing epidemic and public health challenge. Int J Stroke.

[2] Pu L., Wang L., Zhang R., Zhao T., Jiang Y., Han L. (2023). Projected global trends in ischemic stroke incidence, deaths and disability-adjusted life years from 2020 to 2030. Stroke.

[3] Siddiqi T.J., Usman M.S., Shahid I. (2022). Utility of the CHA2DS2-VASc score for predicting ischaemic stroke in patients with or without atrial fibrillation: a systematic review and meta-analysis. Eur J Prev Cardiol.

[4] Saliba W., Gronich N., Barnett-Griness O., Rennert G. (2016). Usefulness of CHADS2 and CHA2DS2-VASc scores in the prediction of new-onset atrial fibrillation: a population-based study. Am J Med.

[5] Lip G.Y.H., Nieuwlaat R., Pisters R., Lane D.A., Crijns H.J.G.M. (2010). Refining clinical risk stratification for predicting stroke and thromboembolism in atrial fibrillation using a novel risk factor-based approach: the euro heart survey on atrial fibrillation. Chest.

[6] Nezami Z., Jujic A., Ohlsson M., Magnusson M., Isholth H.H., Platonov P.G. (2026). Associations between biomarkers and P-wave indices in relation to atrial fibrillation development in heart failure patients. Heart Rhythm.

[7] Wolder L.D., Graff C., Baadsgaard K.H. (2023). Electrocardiographic P terminal force in lead V1, its components, and the association with stroke and atrial fibrillation or flutter. Heart Rhythm.

[8] Segar M.W., Lambeth K., Rosenblatt A. (2026). Electrocardiographic P-wave parameters and lifetime atrial fibrillation risk: a multicohort study. Heart Rhythm.

[9] Weerts J., Țica O., Aranyo J. (2025). Atrial cardiomyopathy: from healthy atria to atrial failure. A clinical consensus statement of the Heart Failure Association of the ESC. Eur J Heart Fail.

[10] Nielsen J.B., Kühl J.T., Pietersen A. (2015). P-wave duration and the risk of atrial fibrillation: results from the Copenhagen ECG Study. Heart Rhythm.

[11] Chang I.C.Y., Austin E., Krishnan B. (2014). Shorter minimum P-wave duration is associated with paroxysmal lone atrial fibrillation. J Electrocardiol.

[12] Jadidi A., Müller-Edenborn B., Chen J. (2018). The duration of the amplified sinus-P-wave identifies presence of left atrial low voltage substrate and predicts outcome after pulmonary vein isolation in patients with persistent atrial fibrillation. JACC Clin Electrophysiol.

[13] Huang T., Nairn D., Chen J. (2023). Structural and electrophysiological determinants of atrial cardiomyopathy identify remodeling discrepancies between paroxysmal and persistent atrial fibrillation. Front Cardiovasc Med.

[14] Huang T., Schurr P., Muller-Edenborn B. (2023). Analysis of the amplified P-wave enables identification of patients with atrial fibrillation during sinus rhythm. Front Cardiovasc Med.

[15] Jagodzinski A., Johansen C., Koch-Gromus U. (2020). Rationale and design of the Hamburg city health study. Eur J Epidemiol.

[16] Zagoridis K., Koutalas E., Intzes S. (2023). P-wave duration and interatrial block as predictors of new-onset atrial fibrillation: a systematic review and meta-analysis. Hellenic J Cardiol.

[17] Müller P., Ivanov V., Kara K. (2017). Total atrial conduction time to predict occult atrial fibrillation after cryptogenic stroke. Clin Res Cardiol.

[18] Masood S., Ashraf S.M.K., Malik M.A., Wahab S. (2023). P-wave indices and left atrial mechanics as predictors of atrial cardiopathy in embolic stroke of undetermined source. Sci Rep.

[19] Kamel H., Soliman E.Z., Heckbert S.R. (2014). P-wave morphology and the risk of incident ischemic stroke in the multi-ethnic study of atherosclerosis. Stroke.

[20] Lin M., Zhu H. (2025). ECG changes and notable markers in ischemic stroke: a systematic review. Heliyon.

[21] Martínez-Sellés M., Elosua R., Ibarrola M. (2020). Advanced interatrial block and P-wave duration are associated with atrial fibrillation and stroke in older adults with heart disease: the BAYES registry. Europace.

[22] Eichenlaub M., Mueller-Edenborn B., Minners J. (2021). Left atrial hypertension, electrical conduction slowing, and mechanical dysfunction – the pathophysiological triad in atrial fibrillation-associated atrial cardiomyopathy. Front Physiol.

