# Supplementary material for: Amplified P-wave duration predicts incident atrial fibrillation in the general population: Results from the Hamburg City Health Study
Source: Heart Rhythm O2. 2026 Apr 2;7(7):1242–9. doi: 10.1016/j.hroo.2026.03.033 (PMC13390079; doi:10.1016/j.hroo.2026.03.033)
Supplement: Supplementary Table [file mmc1.docx]

**Table A.1.** Difference in measurement of automated and amplified P-wave duration

|  | **Amplified PWD** | **Automated**  **PWD** | | **Difference**  **amplified – automated PWD** | | ***P*-value** |  |
| --- | --- | --- | --- | --- | --- | --- | --- |
| Total study population, ms (*n*=2,054) | 123.8±15.2 | 116.3±16.1 | 7.6±16.9 | | **<.001*** | | |
| 5% with lowest amplified PWD, ms (*n*=98) | 97.2±4.5 | 98.5±16.2 | -1.3±15.7 | | .424 | | |
| 5% with lowest automated PWD, ms (*n*=103) | 115.8±22.5 | 80.6±15.0 | 35.3±31.2 | | **<.001*** | | |

*Abbr.: PWD=P-wave Duration.*
