# Supplementary figures and images for: Amplified P-wave duration predicts incident atrial fibrillation in the general population: Results from the Hamburg City Health Study
Source: Heart Rhythm O2. 2026 Apr 2;7(7):1242–9. doi: 10.1016/j.hroo.2026.03.033 (PMC13390079; doi:10.1016/j.hroo.2026.03.033)

**A**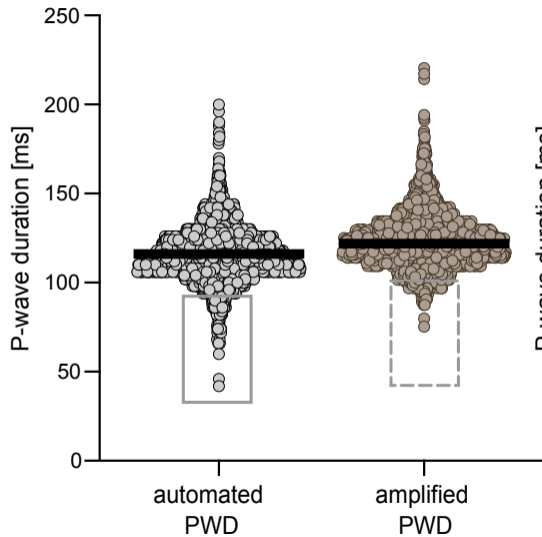**B**

5% with lowest **automated**  
P-wave duration

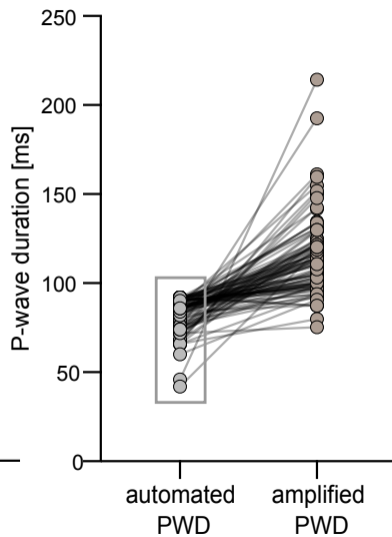**C**

5% with lowest **amplified**  
P-wave duration

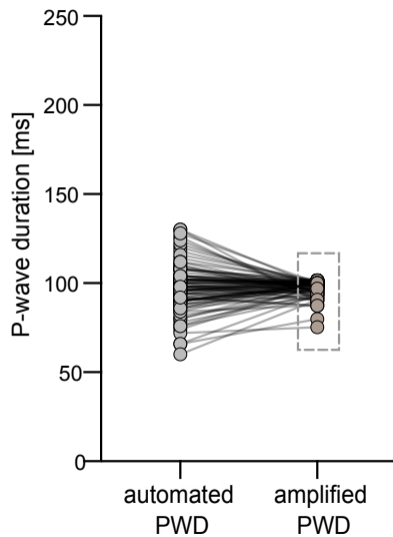

Supplement: Figure A1 [file mmc2.pdf]

**A**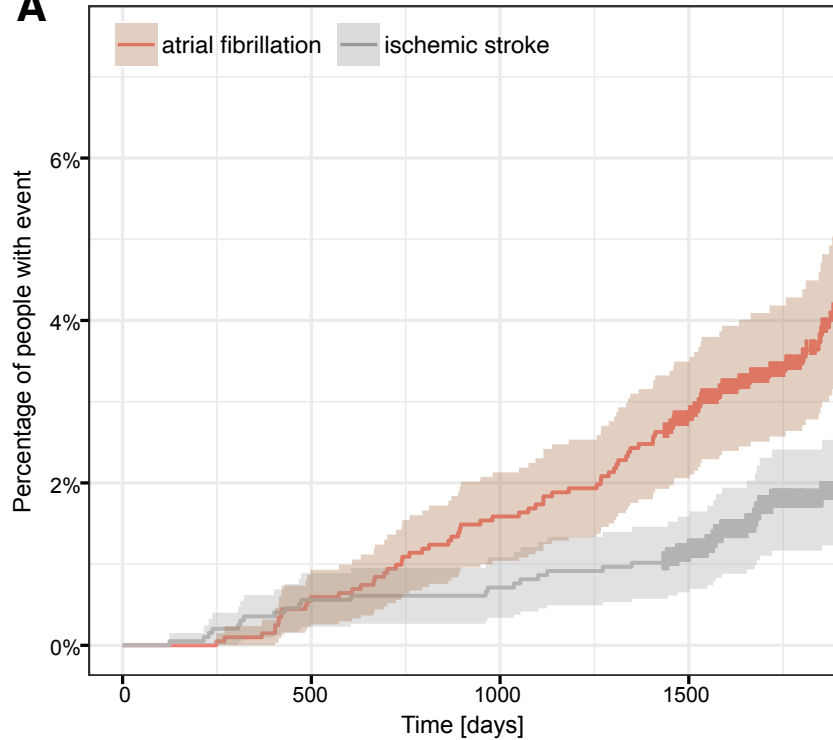**B**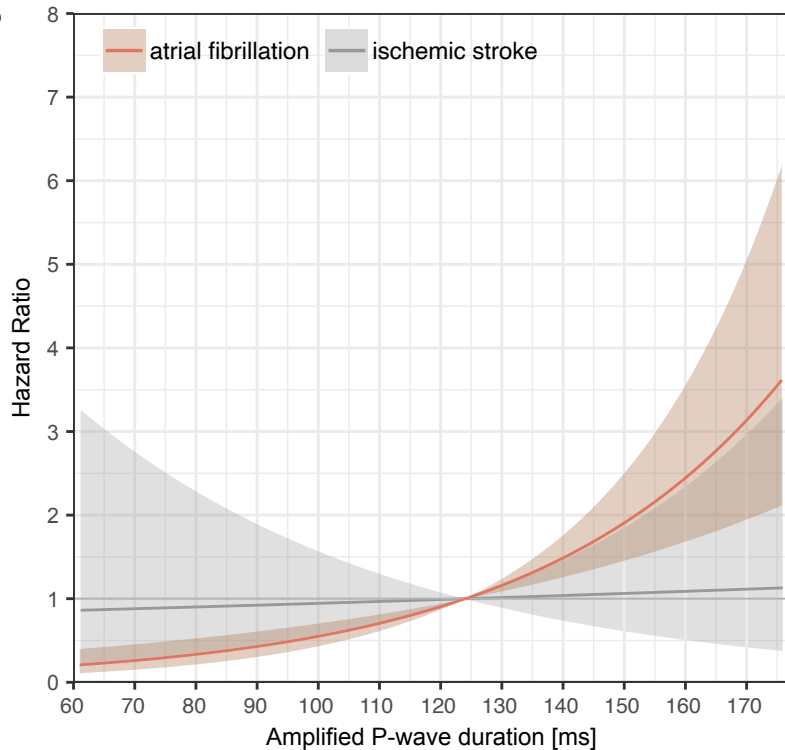

Supplement: Figure A2 [file mmc3.pdf]
